# Injection Drug Use Alters Plasma Regulation of the B Cell Response

**DOI:** 10.3390/cells13121011

**Published:** 2024-06-10

**Authors:** Sanghita Sarkar, Dave D. Hill, Alexander F. Rosenberg, Ellen F. Eaton, Olaf Kutsch, James J. Kobie

**Affiliations:** 1 Infectious Diseases Division, Department of Medicine, University of Alabama at Birmingham, Birmingham, AL 35249, USA; 2 Department of Microbiology, University of Alabama at Birmingham, Birmingham, AL 35249, USA

**Keywords:** B cell, plasma, inflammation, heroin, opioid, injection, germinal center

## Abstract

The opioid epidemic continues to be a major public health issue that includes millions of people who inject drugs (PWID). PWID have increased incidence of serious infections, including HIV as well as metabolic and inflammatory sequelae. We sought to discern the extent of systemic alterations in humoral immunity associated with injection drug use, including alterations in the plasma proteome and its regulation of B cell responsiveness. Comprehensive plasma proteomics analysis of HIV negative/hepatitis C negative individuals with a history of recent injection heroin use was performed using mass spectrometry and ELISA. The effects of plasma from PWID and healthy controls on the in vitro proliferation and transcriptional profile of B cell responses to stimulation were determined by flow cytometry and RNA-Seq. The plasma proteome of PWID was distinct from healthy control individuals, with numerous immune-related analytes significantly altered in PWID, including complement (C3, C5, C9), immunoglobulin (IgD, IgM, kappa light chain), and other inflammatory mediators (CXCL4, LPS binding protein, C-reactive protein). The plasma of PWID suppressed the in vitro proliferation of B cells. Transcriptome analysis indicated that PWID plasma treatment increased B cell receptor and CD40 signaling and shifted B cell differentiation from plasma cell-like toward germinal center B cell-like transcriptional profiles. These results indicate that the systemic inflammatory milieu is substantially altered in PWID and may impact their B cell responses.

## 1. Introduction

The US opioid epidemic, which is in its 4th wave, is dominated by combined opioid and stimulant use, and is an escalating public health crisis with numerous biological and psychological drivers and consequences that are amplified in marginalized populations [1]. Opioid and stimulant use disorders are associated with increased risk and severity of infections and immune dysregulation that can contribute to cardiovascular, kidney, liver, and autoimmune diseases [2,3,4,5]. Opioid-related hospitalizations are ~300 per 100,000 total population [6] and often a result of serious infections (such as hepatitis C, HIV, bacterial endocarditis, and skin and soft-tissue infections), representing a major public health burden. 

Opioid use is linked to the activation of various immune cells, including T cells, B cells, natural killer (NK) cells, neutrophils, monocytes, and macrophages [7,8,9]. Opioids, including heroin, can directly impact the immune system by directly interacting with opioid receptors on immune cells, resulting in decreased T cell proliferation and cytokine production [10] as well as impaired phagocytosis and chemotaxis on macrophages [11]. Mu opioid receptor (MOR) engagement on immune cells triggers the activation of nuclear factor kappa-light-chain-enhancer of activated B cells (NF-κB) and protein kinase C (PKC) ζ [12,13], resulting in the secretion of proinflammatory cytokines [14], impaired Th-1 responses, and compromised antibody production [15,16]. In vitro studies have shown that MOR stimulation induces the production of proinflammatory cytokines [12,13] and cellular activation [17] in peripheral blood mononuclear cells (PBMC). In vivo, the effects of opioids on immune regulation are mediated in part by increasing bacterial and/or fungal translocation, disrupting intestinal homeostasis through alterations in mucosal barrier function [18], mucus secretion [19], and/or bile acid metabolism [20,21]. Notably, there is a convergence of the toll-like receptor 4 (TLR4) and MOR signaling pathways [22,23] that likely contributes to these effects.

In clinical studies, heroin use is associated with elevated biomarkers of chronic inflammation and microbial translocation, independent of HIV infection [24,25]. This association is characterized by increased levels of soluble CD14, LPS-binding protein (LBP), and beta-D-glucan. These changes coincide with the expression of activation markers (CD38 and HLA-DR) on T cells in both tissue and blood, T cell proliferation, and myeloid activation [26].

As B cells and antibodies are essential members of the adaptive immune response, responsible for protection from infectious pathogens, mediating autoimmune diseases, and driving inflammatory processes, the impact of sustained opioid use on their responsiveness may have broad-reaching consequences. In mice, morphine has been shown to inhibit antibody responses [11,27,28]. Although heroin, and recently fentanyl, dominate illicit injection drug use, polydrug use is substantial among people who inject drugs (PWID), including use of cocaine and methamphetamine [29,30], which prevents clear extrapolation of the impact of opiates on B cells in animal models to the complexity of PWID, necessitating a direct and comprehensive assessment of the influence of injection drug use on B cell responsiveness.

We and others have described significant immune alterations in individuals that inject opioids and/or stimulants, including increased systemic inflammation and B cell dysregulation [24,25]. Although the direct immune modulating effects of opioids and stimulants are partially described, primarily from in vitro and animal studies, the drivers of inflammation and B cell dysregulation in PWID are poorly understood and are likely the result of the substances, as well as behavioral and environmental influences. In addition to potentially contributing to the increased incidence of inflammation-mediated co-morbidities, chronic exposure to opioids and stimulants contributes to humoral dysregulation which may limit protection from pathogens, including the ability to generate effective responses to vaccines.

The B cell response is dependent on direct engagement of the B cell receptor (BCR) with its cognate antigen, and the integration of numerous diverse co-stimulatory signals such as TLR agonists, cytokines, chemokines, and T cell help which finely tune the resulting localization, proliferation, differentiation, and specialization of the B cell response. Although general phenotypic alterations in B cells and variations in antibody responses have been reported with injection drug use, opioids, and stimulants, there remains minimal resolution of their impact on responsiveness to BCR engagement and transcriptional programming. Here, we define the impact of injection heroin and cocaine use on the plasma composition and its impact on B cell responsiveness. 

## 2. Materials and Methods

### 2.1. Clinical Samples

Peripheral blood samples were obtained from active PWID recruited through a syringe exchange program in Rochester, NY, USA and healthy control (HC) individuals were recruited at the University of Rochester Medical Center during 2014 and have been previously described [24]. All participants provided signed, written informed consent. The study was conducted in accordance with the Declaration of Helsinki, and all procedures and methods were approved by the University of Rochester Research Subjects Review Board.

### 2.2. DIA-Mass Spectrometry

Plasma samples from healthy and injection drug user donors were analyzed by MS Bioworks (Ann Arbor, MI, USA) for DIA-MS analysis. Briefly, plasma was depleted using the Pierce Top 14 Abundant Depletion Spin Columns kit. Protein was concentrated and buffer exchanged versus water using a 5 KDa MWCO spin filter and quantitated by Qubit fluorometry. Briefly, samples were reduced in DTT followed by alkylation in 15 mM iodoacetamide, trypsin added, and acidified to 0.3% TFA and subjected to solid phase extraction. Briefly, the matrix was activated with 4× additions of 500 μL of 70% acetonitrile and the activated matrix was equilibrated with 4× additions of 500 μL of 0.3% TFA. Samples were added to each well, washed three times with 500 μL of 0.3% TFA, and eluted in 600 μL of 60% acetonitrile in 0.3% TFA. Samples were frozen at −80 °C and lyophilized overnight. Next, 1 μg per sample was analyzed by nano LC/MS with a Waters M-class HPLC system (Waters, Milford, MA, USA) interfaced to a ThermoFisher Exploris 480 (Thermo Fisher Scientific, Waltham, MA, USA). Peptides were loaded on a trapping column and eluted over a 75 μm analytical column at 350 nL/min with a 90 min gradient employed. The mass spectrometer was operated in data-independent mode. Sequentially, full scan MS data (60,000 FWHM resolution) from *m*/*z* 385–1015 was followed by 61 × 10 *m*/*z* precursor isolation windows, another full scan from *m*/*z* 385–1015 was followed by 61 × 10 *m*/*z* windows staggered by 5 *m*/*z*; products were acquired at 15,000 FWHM resolution. DIA data were analyzed using Scaffold DIA 3.2.1 (Proteome Software, Portland, OR, USA).

### 2.3. ELISA

Plasma IL-6 was measured by the HS Human IL-6 immunoassay kit (R&D Systems, HS600C, Minneapolis, MN, USA), CRP was measured by the Human C-Reactive Protein/CRP kit (R&D Systems, DCRP00B). CXCL4 was measured using the Human CXCL4 ELISA (R&D Systems, DY795). LPS binding protein (LBP) and sgp130 in plasma was measured using the Human LBP (R&D Systems, DY870-05) and Human sgp130 ELISA (R&D Systems, DY228) kits respectively.

### 2.4. In Vitro Lymphocyte Proliferation Assays

PBMCs were thawed and centrifuged at 300× *g* for 5 min. Cells were labeled according to the manufacturer’s protocol with 1 mL of CFSE (cat # C34554, Invitrogen, Waltham, MA, USA) fluorescent dye and incubated at 37 °C for 20 min. Cells were centrifuged at 300× *g* for 5 min and resuspended in fresh pre-warmed complete medium before proceeding with cell stimulation assays. Fifty thousand CFSE labeled PBMCs were cultured in RPMI 1640 supplemented with 10% FBS and 1× antibiotic-antimycotic, in a total volume of 200 mL in 96 well round bottom plates. Cells were stimulated with 40 ng/mL IL-2 (cat # 200-02, Peprotech, Cranbury, NJ, USA), 4 mg/mL sCD40L (cat # 310-02, Peprotech), and 40 mg/mL anti-human IgM (cat # 109-005-043, Jackson ImmunoResearch, West Baltimore, PA, USA) in the presence of HC or PWID plasma at a dilution of 1:50. Cells were cultured at 37 °C in 5% CO_2_ for 96 h. Cultured cells were then stained with live/dead blue stain (cat # L23105, Invitrogen) for 15 min, followed by washing cells at 300× *g* for 5 min. Cells were resuspended and labeled with anti-CD4-BB790 (clone L200, cat # custom, BD Biosciences, Franklin Lakes, NJ, USA), anti-CD19-APC-Cy7 (clone SJ25C1, cat # 557791, BD Pharmingen), anti-CD20-Alexa-fluor 700 (clone 2H7, cat # 302322, Biolegend, San Diego, CA, USA), anti-CD11c-BV421 (clone 3.9, cat # 565807, BD Horizon, Franklin Lakes, NJ, USA), anti-IgM-BV605 (clone G20-127, cat # 562977, BD Horizon), anti-CD27-BV650 (clone O323, cat # 302828, Biolegend), anti-CD86-BV711 (clone FUN-1, cat # 563158, BD Horizon), anti-CD69-BUV395 (clone FN50, cat # 564364, BD Horizon), anti-CD8-BUV496 (clone RPA-T8, cat # 564805, BD Horizon), anti-CD95-BUV737 (clone DX2, cat # 564710, BD Horizon), anti-CD151-PE (clone 14A2.H1, cat # 556057, BD Pharmingen), and anti-CD21-PECy-5 (clone B-ly4, cat # 551064, BD Pharmingen) for 1 h on ice then washed twice, fixed, and analyzed on BD FACS symphony (A3). FCS files were analyzed with FlowJo software v10 (FlowJo, Ashland, OR, USA). Briefly, total PBMCs were gated on lymphocytes using FSC and SSC. Live/dead stain and anti-CD4 were used to eliminate dead cells and CD4+ T cells, respectively, and total B cells were identified as CD19+CD20+. Total B cells were further gated down to various B cell subsets and were identified as naïve (CD21+CD27-), activated memory (CD21-CD27+), tissue-like memory (TLM) (CD21-CD27-), and resting memory (CD21+CD27+) B cells. FlowJo software’s proliferation tool was used for calculation of expansion and division indexes [31].

### 2.5. RNA Sequencing

For RNA sequencing, B cells were sorted from PBMC. RNA extractions were performed followed by a library preparation. Ultra-low input RNA-Seq was performed at Azenta NGS laboratory with Illumina HiSeq (Illumina, San Diego, CA, USA), 2 × 150 bp with ~350 M reads. FASTQ reads were first run through FastQC (version 0.11) for quality control and then processed by TrimGalore! (version 0.44) [32,33] to trim adapters and low-quality reads. Trimmed FASTQ files were mapped to the GRCm39 mouse genome using STAR (version 2.5) [34] and output to sorted-by-coordinates bam files. Sorted bam files were processed by HTSeq-count (version 0.6.1) [35] with the parameter “-s no” to generate count data. The count matrix was analyzed using the R package EdgeR v4.2.0 [36]. Genes with > 1 counts per million in at least 3 samples were retained. For differential expression analysis, glmQLFit was applied to the count data and glmQLFTest with the contrast STNeg-over-STPos was used. Genes with an FDR value of <0.05 and an absolute value of log2 fold change ≥ 1 were defined as differentially expressed genes (DEGs). Gene Set Enrichment Analysis (GSEA) [37] was performed using the C7 Immunological Signature Gene Sets [38]. Ingenuity Pathway Analysis (IPA, QIAGEN Digital Insights, Hilden, Germany) was performed to identify upstream regulators and downstream targets. 

### 2.6. Statistical Analysis

Univariate analysis of individual features was performed by the two-tailed Student’s *t*-test to compare groups using Prism 10.0 software (GraphPad Software, La Jolla, CA, USA).

## 3. Results

### 3.1. Cohort Description

This cohort was previously described [24] and includes a group of young adult HIV negative and hepatitis C negative individuals with active injection heroin use (PWID; *n* = 19) recruited through an urban syringe exchange program and a group of healthy control (HC) individuals (*n* = 19) with similar demographic composition to the PWID group. Female sex assigned at birth was 32% and 42% for PWID and HC, respectively. The average age was 28.4 and 24.3 years old for PWID and HC, respectively. The majority (56%) of the PWID reported daily heroin use for the preceding 3 months, and combined use of heroin and cocaine was dominant, with 89% of PWID reporting also injecting cocaine within the preceding 30 days [24]. 

### 3.2. Distinct Plasma Proteomics Profile of PWID

**Figure 1:**
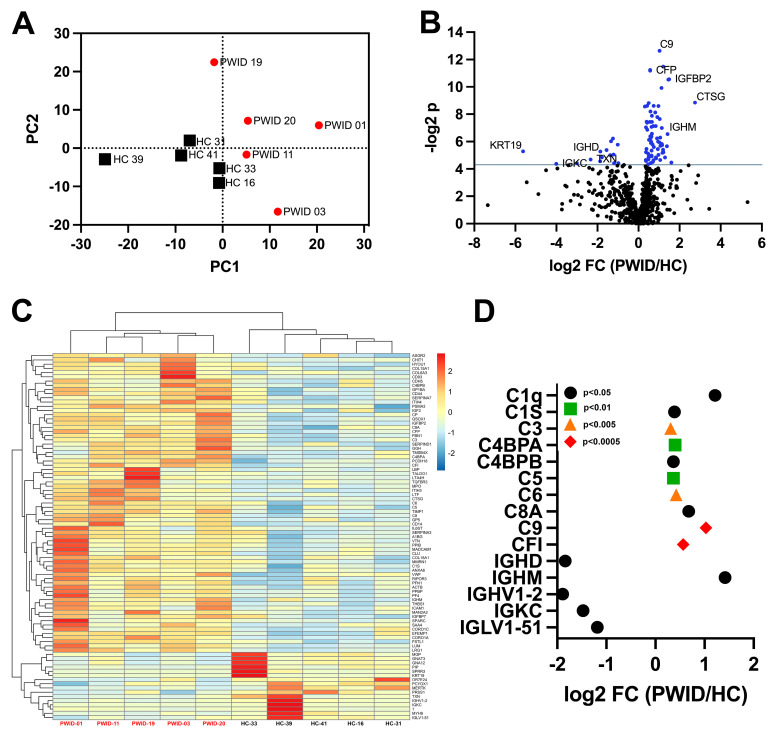
Distinct plasma proteome of PWID. Plasma from PWID (*n* = 5) and HC (*n* = 5) was analyzed by DIA-MS. (**A**) PCA of plasma profiles based on all measured proteins (*n* = 666). (**B**) Identification of significantly differentially expressed proteins, blue indicates *p* < 0.05. (**C**) Relative expression of all differentially expressed proteins. (**D**) Fold-change (log_2_FC) of select complement and immunoglobulin-related differentially expressed proteins.

To discern potential differences in the composition of plasma from PWID that may impact B cell responsiveness, a mass-spectrometry-based data-independent acquisition (DIA-MS) approach was used to determine the proteomics profile of PWID plasma. Specifically, plasma from five PWID whose comprehensive cellular and plasma immune profile has previously been described as being distinct from HC [24] was assessed in comparison to five representative HC. The abundance of 721 proteins was determined, revealing the plasma proteomes of PWID were highly distinct from HC (Figure 1A) and 86 proteins were significantly differently expressed (Figure 1B,C).

Many inflammatory factors were significantly elevated, including sCD14, sCD44, and LPS binding protein (LBP), consistent with previous reports [39,40,41]. Additionally, many complement components and platelet/coagulation-related factors including pro-platelet basic protein (PPBP) and platelet factor 4 (PF4/CXCL4), were elevated that have not been previously appreciated. IgM was significantly elevated in PWID, while IgD and kappa light chain were significantly decreased (Figure 1D). A significant decrease in immunoglobulin utilizing the VH1-2 heavy chain variable region was also evident in PWID, which is notable as it has previously been reported as enriched in a subset of HIV-specific broadly neutralizing antibodies targeting the conserved CD4 binding site [42]. 

**Figure 2:**
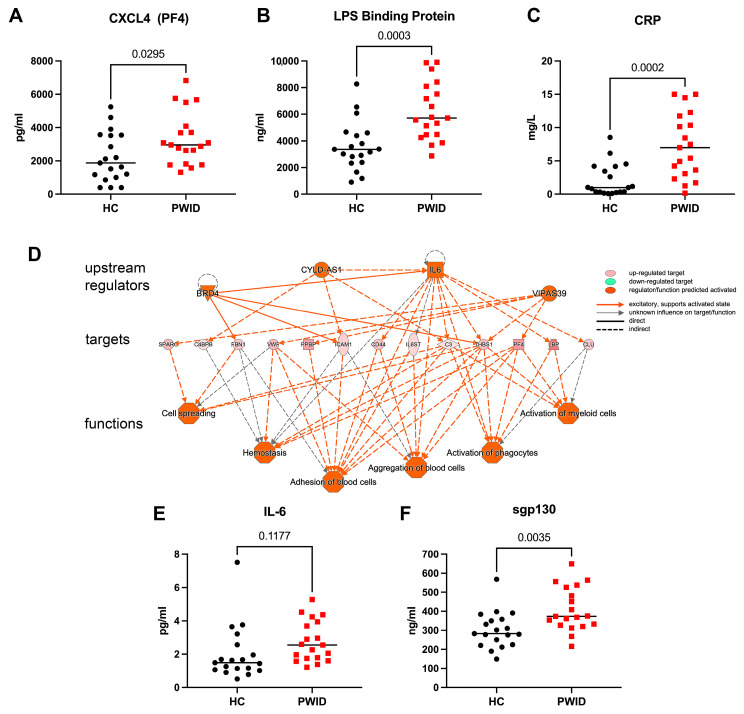
Elevated inflammatory mediators in PWID. Plasma from PWID (*n* = 19) and HC (*n* = 19) was evaluated by ELISA to determine abundance of CXCL4 (**A**), LPS binding protein (LPS BP) (**B**), C-reactive protein (CRP) (**C**), IL-6 (**E**), and sgp130 (**F**). Each symbol represents a unique individual. Upstream and downstream analysis using Ingenuity Pathway Analysis of DIA-MS-determined proteome (**D**). Significance determined by *t*-test.

Several analytes that varied in PWID based on the DIA-MS analysis were measured by ELISA in the full cohort. PF4 (CXLC4), the platelet derived chemokine, which is also targeted by autoantibodies in heparin-induced thrombocytopenia [43,44], was significantly increased in PWID (Figure 2A). LBP, integral for TLR4 signaling [45,46], was significantly increased in PWID (Figure 2B), consistent with previous reports of increased plasma LPS in PWID, and suggestive of decreased gut integrity [40]. C-reactive protein (CRP) was significantly increased in PWID, with 58% of PWID having CRP > 5.0 mg/L (Figure 2C), indicative of increased systemic inflammation and consistent with previous reports [25]. Upstream and downstream pathways analysis based on DIA-MS analysis suggested IL-6 was a major driver of the altered proteome observed in the PWID plasma (Figure 2D), and increased IL-6 and soluble gp130 were evident among PWID in the full cohort (Figure 2E,F). These results are consistent with a chronic systemic inflammatory phenotype in PWID that may contribute to the observed B cell dysregulation.

### 3.3. Inhibition of B Cell Proliferation by PWID Plasma

**Figure 3:**
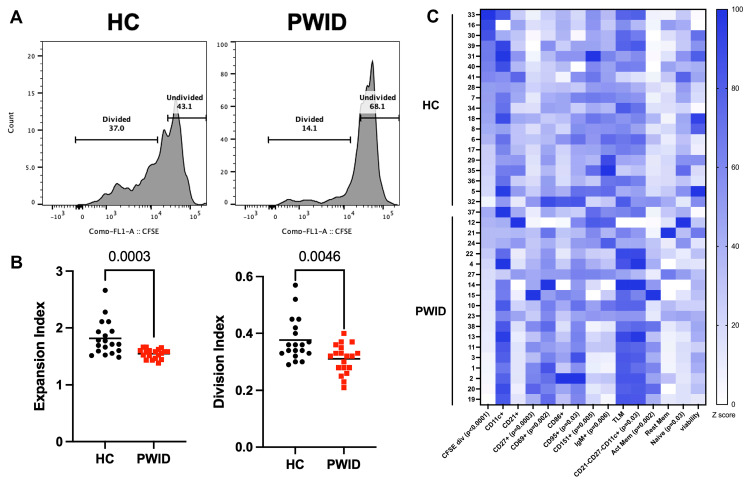
Inhibition of B cell proliferation by PWID plasma. CFSE-labeled PBMC from a healthy control was cultured in IL-2, CD40L, and α-IgM for 4 d in the presence of HC (*n* = 19) or PWID (*n* = 19) plasma (1:50) samples and analyzed by flow cytometry. (**A**) Representative histograms gated on live CD19+ B cells. (**B**) Metrics of B cell proliferation. Each symbol represents an individual. (**C**) Frequency of divided B cells (CFSE div) and divided B cells expressing indicated marker or subset; naïve (CD21+CD27-), activated memory (CD21-CD27+), TLM (CD21-CD27-) and resting memory (CD21+CD27+) normalized to lowest frequency for each parameter. Significance determined by *t*-test.

Observing the altered profile of plasma analytes in PWID, we next sought to directly determine the influence of PWID plasma on the activation and proliferation of B cells. Total PBMC from a healthy control was used to preserve the ability of B cells to respond in a complex environment with the presence of other lymphocytes and stimulated with α-IgM and CD40L as a surrogate for a primary B cell response in the presence of plasma from PWID or HC. B cells stimulated in the presence of PWID plasma had a reduced ability to proliferate, with a significant reduction in their expansion and division indices compared to B cells stimulated in the presence of HC plasma (Figure 3A,B). Among the divided B cells, PWID plasma treatment resulted in significantly increased frequencies of CD27+, CD69+, and CD95+ cells compared to HC plasma treatment (Figure 3C), suggestive of increased B cell activation. Decreased IgM+ and CD151+ cells among the divided B cells from PWID plasma treatment as compared to HC plasma treatment were evident, which may suggest an impact on differentiation. A significantly lower frequency of B cells with naïve (CD21+CD27-) and an increased frequency of activated memory (CD21-CD27+) and atypical memory (CD21-CD27-CD11c+) phenotypes were evident among the divided cells with PWID plasma treatment. The naïve B cells from PWID plasma treatment had increased CD69 expression (Supplementary Figure S1). These results suggest that PWID plasma promotes robust B cell activation yet constrains B cell proliferation. 

### 3.4. PWID Plasma Alters B Cell Transcriptional Response to Activation

**Figure 4:**
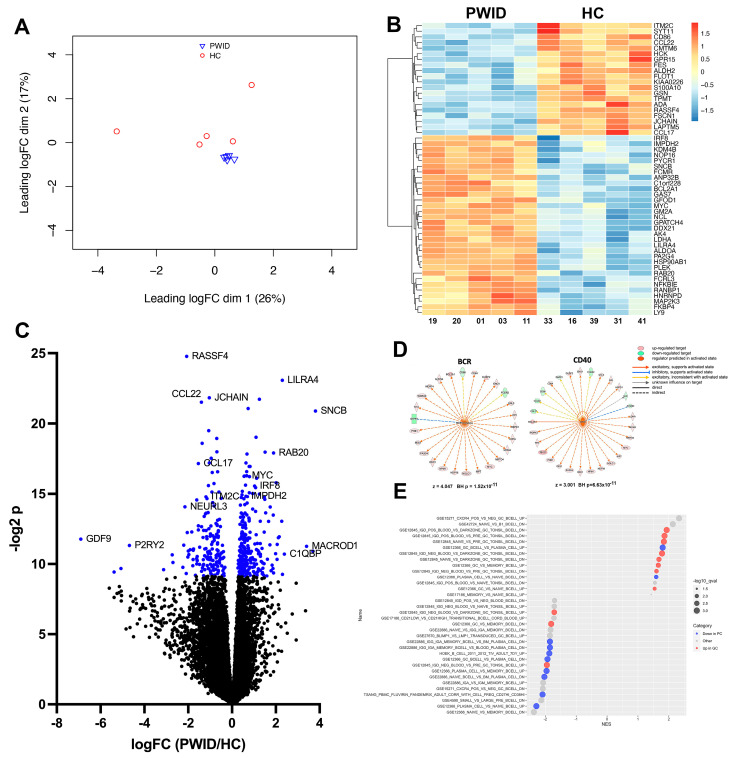
PWID plasma alters B cell response to stimulation. PBMC from a healthy control was cultured in IL-2, CD40L, and α-IgM for 4 d in the presence of HC (*n* = 5) or PWID (*n* = 5) plasma (1:50), and B cells were then isolated and analyzed by RNA-Seq. (**A**) PCA plot of the PWID and HC samples. (**B**) Heatmap of the differentially expressed genes. (**C**) Volcano plot of RNA-Seq data. Blue indicates FDR < 0.05. (**D**) Upstream regulators of BCR and CD40 and downstream targets that overlap with DEGs as determined using Ingenuity Pathway Analysis. For targets, red is upregulated and green is downregulated. For regulators (center), orange is predicted to be activated. Z-score of activation and Benjamini–Hochberg-corrected *p*-value of overlap are indicated. (**E**) Enriched gene sets using GSEA with the C7 Immunological Signature Gene Sets. X-axis is normalized enrichment score, y-axis is the gene set, symbol size is proportional to –log10 of the enrichment FDR q-value, and color indicates direction of change in the gene set. Gene sets comparing plasma cells (PC) and germinal center B cells (GC) are shown.

To gain insight into the molecular basis for the ability of PWID plasma to alter B cell responsiveness, the transcriptional profile of B cells stimulated with α-IgM and CD40L in the presence of PWID plasma was assessed. The transcriptome of PWID plasma-treated B cells was very similar, with limited inter-group heterogeneity. In contrast, HC plasma-treated B cells had more apparent inter-group diversity (Figure 4A). Among the notable differentially expressed genes (DEGs), *ILT7* (*LILRA4*), a regulatory receptor that mediates the suppression of type I IFNs and TNF-α in response to TLR7 and TLR9 ligands [47], was increased in PWID-treated B cells (logFc = 2.30, q = 6.64 × 10^−4^). *CCL22* (logFc = −1.39, q = 7.79 × 10^−4^) and *CCL17* (logFc = −1.54, q = 4.25 × 10^−3^), which bind *CCR4* on T cells to regulate the affinity maturation process during the germinal center reaction [48], were decreased in PWID-treated B cells as compared to HC (Figure 4B,C). *ITM2C*, which is highly and selectively expressed in antibody secreting cells [49,50], was decreased (logFc = −1.19, q = 9.06 × 10^−3^) with PWID treatment, while *IMPDH2* (logFc = 0.64, q = 9.06 × 10^−3^) and *IRF8* (logFc = 1.03, q = 7.16 × 10^−3^), which are highly associated with germinal center B cells and the suppression of plasma cell (PC) development [51,52,53], were increased with PWID treatment. Using upstream analysis, upstream B cell receptor (BCR) (z = 4.407) and CD40 (z = 3.001) were found to be significantly enriched in upstream regulators predicted to be activated in PWID plasma-treated B cells (Figure 4D), suggesting that inhibition of B cell proliferation by PWID plasma is not a consequence of diminished BCR and CD40 responsiveness. Gene set enrichment analysis (GSEA) of human B cell signatures indicated PWID plasma treatment promoted more germinal center (GC) B cell and less PC-like transcriptional signatures compare to HC plasma-treated B cells (Figure 4E). These results suggest that PWID plasma increases BCR and CD40 responsiveness and promotes more GC-like rather than PC-like development. 

## 4. Discussion

The combined opioid and stimulant use epidemic, which represents the 4th wave of the opioid crisis, is an escalating public health emergency with numerous biological and psychological drivers and consequences. While significant immune alterations in PWID, including increased systemic inflammation and lymphocyte dysregulation, have been reported and are likely to contribute to increased risk and severity of infections and inflammatory co-morbidities, including cardiovascular, kidney, liver, and autoimmune diseases, the relationship between this heightened inflammatory milieu and B cell function in PWID remains poorly defined.

Our study sought to profile the plasma of PWID and its functional consequence on B cells. Global plasma proteomics analysis indicated PWID were highly distinct from HC, with numerous inflammatory factors elevated in PWID. This is suggestive of a chronic systemic inflammatory phenotype in PWID, consistent with systemic inflammation in PWID observed in previous studies [54,55,56,57,58] and may contribute to co-morbidities as chronic inflammation is associated with cardiovascular disease, kidney disease, accelerated aging, and neurodegenerative diseases, all of which are increased among PWID [5,59,60,61,62,63]. Elevated LBP in the plasma of PWID suggests increased bacterial exposure and increased gut permeability and coincides with previous findings [24]. Numerous indications of altered IL-6 homeostasis in PWID were apparent, including significantly elevated complement components and CRP, which are synthesized by hepatocytes in response to IL-6 [64], and increased IL-6 and the IL-6 signal transducing membrane gp130 [65]. The elevation of platelet and coagulation-related factors such as PF4 (CXCL4), also regulated by IL-6 [66], may be related to the increased incidence of thrombocytopenia among PWID [67,68,69]. These results suggest that IL-6 targeted interventions to mitigate the chronic inflammatory state associated with injection drug use should be studied.

As an initial step for evaluating the potential impact of the altered plasma profile of PWID on B cell responses, we intentionally used a composite culture system of total PBMC to enable both direct and indirect influences of plasma analytes on B cell proliferation and differentiation. B cells stimulated with plasma from PWID showed reduced ability to proliferate, with significant reduction in their expansion and division indices. However, among the divided B cells, PWID plasma treatment resulted in significantly increased frequencies of CD27+, CD69+, and CD95+ cells and decreased IgM+ cells as compared to HC plasma treatment, suggesting that PWID plasma is promoting robust B cell activation. Transcriptional analysis confirmed this, with significantly greater BCR and CD40 signaling activation with PWID plasma treatment. This restrained proliferation with PWID plasma treatment was associated with a more germinal center-like transcriptional profile, compared to a more typical plasma cell-like profile evident with HC plasma treatment. The relationship between regulation of proliferation and developmental programming by PWID plasma, and particular plasma factor(s) mediating this process warrants further evaluation.

A limitation of this study is that the particular plasma analyte(s) that are most consequential for the PWID effect on B cell response were not defined, and that this in vitro system may not fully reflect the chronic exposure to the plasma analytes that occurs in vivo. However, this study does clearly demonstrate the altered and heightened systemic inflammatory profile of PWID plasma and its potential functional consequences to B cell responses. Given the elevated inflammation and immune dysregulation in PWID, now the field must advance to the development of interventions that can decrease inflammatory and infection-related co-morbidities in these individuals.

## 5. Conclusions

These results indicate the systemic inflammatory milieu is substantially altered in PWID and may impact their B cell responses.

## Data Availability

The raw data supporting the conclusions of this article will be made available by the authors on request.

## Supplementary Materials

The following supporting information can be downloaded at: https://www.mdpi.com/article/10.3390/cells13121011/s1, Figure S1: Expression of CD69, CD95, and CD151 on major B cell subsets.
