# Supplementary material for: Injection Drug Use Alters Plasma Regulation of the B Cell Response
Source: Cells. 2024 Jun 10;13(12):1011. doi: 10.3390/cells13121011 (PMC11202061; doi:10.3390/cells13121011)
Supplement: Supplementary file 1 [file cells-13-01011-s001.zip › cells-3008455-supplementary.pdf]

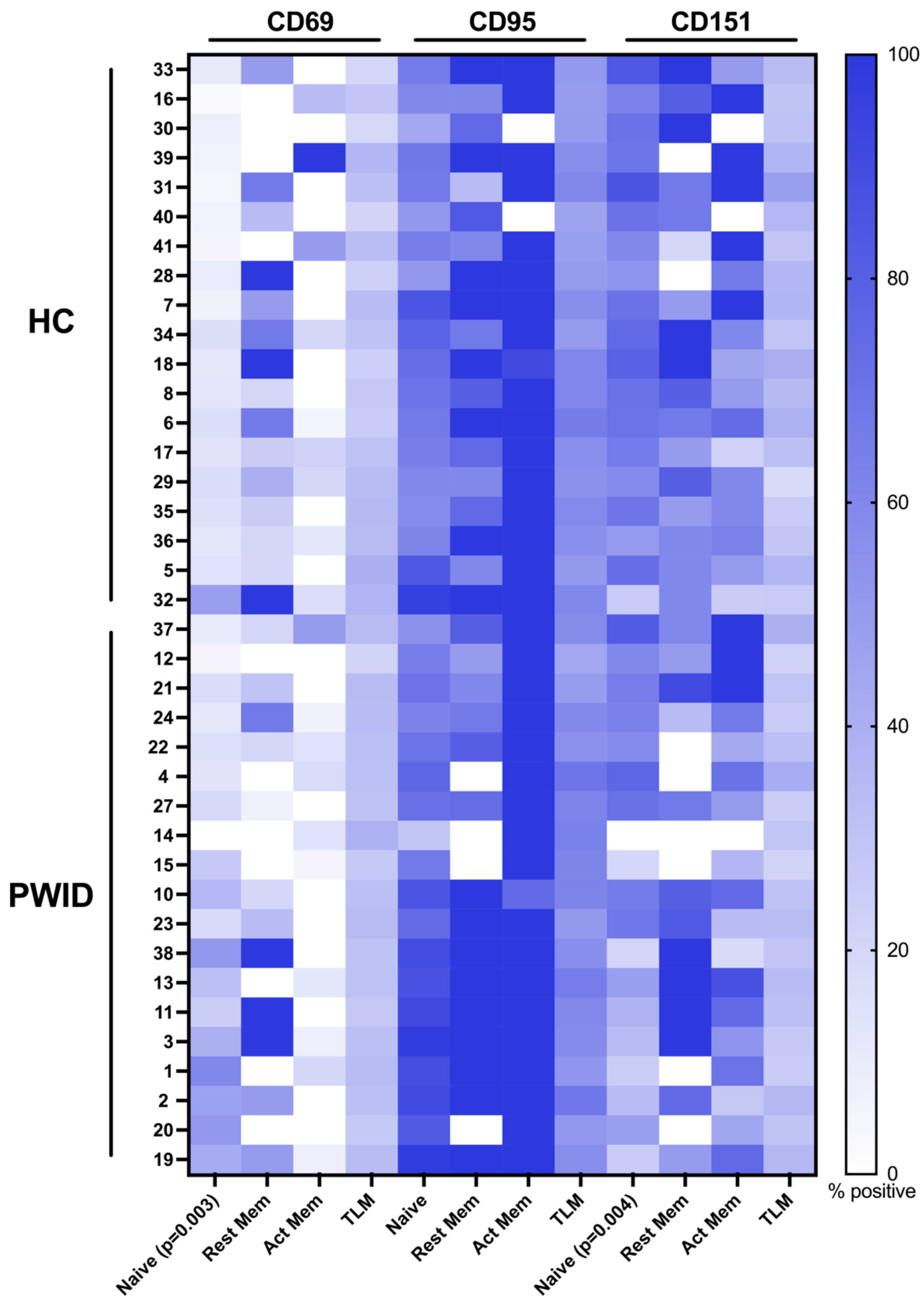

Supplemental Figure 1. Expression of CD69, CD95, and CD151 on major B cell subsets. Frequency of CD69, CD95, and CD151 expressing B cells expressing among subsets of divided B cells; naïve (CD21+CD27-), activated memory (CD21-CD27+), TLM (CD21-CD27-) and resting memory (CD21+CD27+). Significance determined by t-test.
